# Deficits in Emotional Perception–Related Motor Cortical Excitability in Individuals With Trait Anxiety: A Transcranial Magnetic Stimulation Study

**DOI:** 10.1155/2024/5532347

**Published:** 2024-09-24

**Authors:** Hui Liu, Linqi Wang, Xiaoying Tan, Jian Zhang, Xue Xia

**Affiliations:** ^1^Shanghai Punan Hospital of Pudong New District, Shanghai, China; ^2^School of Psychology, Shanghai University of Sport, Shanghai, China; ^3^Faculty of Health Sciences and Sports, Macao Polytechnic University, Rua de Luis Gonzaga Gomes, Macau, Macao S.A.R., China; ^4^Qingdao Hospital, University of Health and Rehabilitation Sciences (Qingdao Municipal Hospital), Qingdao, China; ^5^School of Social Development and Health Management, University of Health and Rehabilitation Sciences, Qingdao, China

**Keywords:** emotional perception, motor cortical excitability, motor-evoked potential, trait anxiety, transcranial magnetic stimulation

## Abstract

Abnormal emotional perception may contribute to emotional dysfunction in individuals with anxiety. This study explored the progression of impaired emotional perception with the deepening of anxiety in individuals with nonclinical trait anxiety, by measuring the motor cortical excitability associated with emotional perception. In total, 87 participants were assigned to a high trait anxiety (*n* = 27), moderate trait anxiety (*n* = 30), or low trait anxiety (*n* = 30) group. Transcranial magnetic stimulation was applied to the right primary motor cortex at 150 ms or 300 ms after the onset of positive, negative, or neutral images, while participants performed an emotion recognition task, and motor-evoked potentials (MEPs) were collected. For participants with low trait anxiety, MEP amplitudes were significantly higher for both negative and positive stimuli than for neutral stimuli. Participants with moderate trait anxiety showed significantly higher MEP amplitudes only for negative stimuli. Participants with high trait anxiety showed no significant difference in MEP amplitudes for positive, negative, and neutral stimuli. Trait anxiety score was negatively correlated with MEP amplitude: For higher trait anxiety scores, MEP amplitudes were correlated with lower emotional perception of positive and negative stimuli. Findings suggest that anxiety impairs emotional perception–related motor cortical excitability, starting with decreased motor cortical excitability responses to positive information and progressing to negative information as anxiety levels increase.

## 1. Introduction

Anxiety is classified as a normal reaction; however, it can become pathological when exceeding specific thresholds, including for conditions such as anxiety disorder, in which an individual experiences fear and excessive worry that interfere with normal daily functioning [1]. Anxiety manifests as trait anxiety, with lifelong patterns that form personality traits and are relatively stable over time, or state anxiety, with acute situation-driven episodes and fluctuating over time [2]. People with anxiety disorders have abnormal emotional perception [3, 4], which may contribute to emotional dysfunction among those with high anxiety, resulting in a reduced ability to label, express, and understand specific emotional states [5, 6]. However, it is unclear how impaired emotional perception progresses with the deepening of trait anxiety in nonclinical populations.

Emotional perception can dynamically drive corticospinal excitability even without real movement output, with an early increase in excitability when perceiving either a positive or negative stimulus indicative of relatively rapid preparation to initiate an approach action or a fight–flight-freeze action, respectively [7–12]. It has been reported that individuals with anxiety show abnormal motor cortical excitability [13, 14], and anxiety-related disorders are associated with a more blunted pattern of physiological reactivity, characterized by hyporeactivity in response to stimuli varying in threat salience [15]. Emotional perception–related cortical excitability may be considered a marker of physiological change in the motor system corresponding to emotion processing [10]. Hence, it could be a promising approach to track changes in emotional perception–related cortical excitability along with the different levels of nonclinical trait anxiety in order to observe the progression of impaired emotional perception.

Transcranial magnetic stimulation (TMS) offers both precise temporal and spatial resolution, making it a valuable tool for investigating motor excitability by the measurement of motor-evoked potentials (MEPs) recorded from a contralateral target muscle [16]. Temporal changes in cortical excitability related to emotional perception can be studied by using single-pulse TMS on the primary motor cortex (M1) while viewing images that evoke emotions [17, 18]. Researchers have identified two separate stages of motor cortex activity: an early attention phase at 150 ms after the onset of an image that corresponds to action preparation and a late evaluation phase at 300 ms that corresponds to action regulation [19]. Motor excitability lateralization during emotional perception has been detected with single-pulse TMS, emphasizing that the right hemisphere is involved in rapidly and temporarily enhancing responses to emotional stimuli, and importantly, that emotion recognition engages the right M1 to a greater extent than the left M1 [20, 21].

Here, we explored how emotional perception–related cortical excitability was affected by anxiety level in a nonclinical sample. Single-pulse TMS was applied over the right M1 at either 150 or 300 ms after stimulus onset during an emotion recognition task that asked participants to recognize the type of emotional images. Participants were individuals with nonclinical high trait anxiety (HTA), moderate trait anxiety (MTA), or low trait anxiety (LTA). We hypothesized that participants in the LTA group would show larger MEP amplitudes during the presentation of an emotional stimulus than during the presentation of a neutral stimulus, whereas individuals in the MTA and HTA groups would show deficits in perceiving emotional stimuli from neutral stimuli, manifested by decreased MEP amplitudes during the presentation of the emotional stimulus.

## 2. Methods

### 2.1. Participants

The goal of this recruitment was to choose three sets of participants with varying levels of nonclinical anxiety (HTA, MTA, and LTA). Therefore, 362 students at Shanghai University of Sport were selected based on their scores on the trait section of the State-Trait Anxiety Inventory [2, 22]. Students whose STAI scores falling within the top 20% (considered HTA in this study), or the middle 20% (MTA), or bottom 20% (LTA) of the distribution were invited to participate in the study. A total of 92 students in the predesignated anxiety categories responded to the invitation. Five students requiring very high stimulation intensities were excluded, leaving 87 participants for data collection. There were 27 participants categorized as the HTA group, 30 participants as the MTA group, and 30 participants as the LTA group. Participant demographics are provided in the Results section.

Each participant involved were right-handed and had either normal vision or corrected-to-normal vision. The inclusion criteria were no history or physician diagnosis of psychiatric or neurologic disorders and no contraindication to TMS [23]. This study was carried out following the Declaration of Helsinki and approved by the Ethics Committee of Shanghai University of Sport (ethics approval number: 102772021RT077). Written informed consent was provided by all participants prior to the study.

### 2.2. TMS

Single-pulse TMS was administered to the right M1 via a 70-mm coil in the shape of a figure eight, powered by a Magstim 200 stimulator (Magstim Company, Dyfed, UK). The coil was placed tangentially to the scalp at a 45° angle to the midline to induce a posterior–anterior current flow across the central sulcus. The motor hotspot is the best location to generate the highest MEP in the specific muscle using a slightly above-threshold stimulation [24]. Single-pulse TMS was administered to the right M1 either 150 or 300 ms following the image onset with an intensity set at 120% of the resting motor threshold (RMT). The RMT was determined as the minimum TMS intensity needed to produce MEPs exceeding 50 μV in a minimum of five out of ten trials while ensuring the muscle is fully relaxed.

### 2.3. Electromyographic Recording

Electromyography (EMG) signals were captured from the left first dorsal interosseous muscle (FDI) using a pair of 9-mm Ag–AgCl electrodes and a ground electrode affixed with a muscle belly–tendon distribution. The FDI was chosen because of the hypothesis that activation of the fight/flight response induced by negative stimuli, including during passive viewing of negative stimuli, is responsible for increased excitability of the hand representation area in M1, a brain structure involved in the detection and reaction to danger that is constantly active to prepare the organism for a rapid action crucial for survival [25]. EMG signals were amplified (×1000), band-pass filtering within the range of 20 Hz to 2.5 kHz, digitally sampled at a rate of 5 kHz using a Micro 1401 device (Cambridge Electronics Design, Cambridge, UK), and were then saved on a computer for offline analysis using SIGNAL 6.0 software. Participants were instructed to sit in an upright position in a chair and maintain open eyes, except for blinking, throughout the experimental session.

### 2.4. Emotional Images

Sixty images were chosen from the International Affective Picture System (IAPS) [26], consisting of 20 images of positive emotions (average valence: 6.3 ± 0.3, average arousal: 5.3 ± 0.3), 20 images of neutral emotions (average valence: 5.0 ± 0.2, average arousal: 4.0 ± 0.9), and 20 images of negative emotions (average valence: 4.1 ± 0.4, average arousal: 5.2 ± 0.2). A previous study has found that IAPS images and emotional faces may activate similar brain regions, but they may also activate different brain regions. This finding may be due to the novelty and complexity of IAPS images compared with emotional faces [27]. Facial expressions/body postures, which may be viewed as relatively unchanging stimuli with consistent features (i.e., facial features of eyes, nose, and mouth and body features of hands and legs), are able to be processed relatively automatically, in contrast to IAPS images, which are composed of complex contextual scenes that are often unique and novel. Viewing the IAPS images may cause sustained activation for all IAPS image conditions (including neutral images) and thus may enable detection of the differences between each emotion type. Hence, IAPS images were used in this study. One-way repeated-measures analysis of variance (RM ANOVA) was conducted separately for the valence and arousal, and both showed significant main effects of image type (valence: *F*_ (2, 38)_ = 285.317, *p* < 0.001, *η*_p_^2^ = 0.938; arousal: *F*_ (2, 38)_ = 48.961, *p* < 0.001, *η*_p_^2^ = 0.720). Post hoc analysis showed significant differences in the emotional valence between each of any two image types (*p* values <0.001), and the arousal associated with either positive or negative images was significantly larger than that for neutral images (*p* values <0.001), with no significant difference in arousal between positive and negative images (*p*=0.902).

### 2.5. Emotion Recognition Task

**Figure 1:**
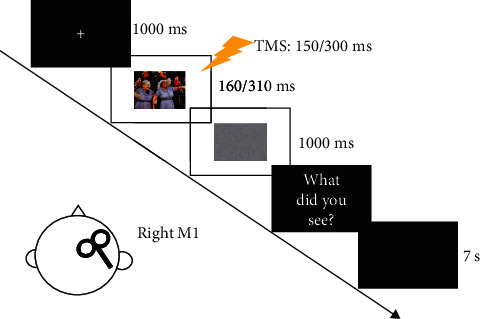
Procedure for the emotion recognition task.

Before the emotion recognition task started, a baseline was established by collecting 10 trials at rest with a TMS intensity of 120% RMT. MATLAB software was utilized to program the procedure for managing the emotion recognition task and initiating TMS (Figure 1). For the emotion recognition task, individuals were shown an image and instructed to classify it as either positive, negative, or neutral [20]. In total, 60 trials, with all images appearing randomly once, were separated into two blocks. Images were presented on a 21.5-inch monitor, and the distance between a participant's forehead and the screen was 60 cm. Each trial was started with a fixation point displayed in the middle of the monitor (for 1000 ms). It was replaced by an image displayed for either 160 or 310 ms (50% probability for each and randomly distributed). In the trials with an image that was displayed for 160 ms, the TMS was administrated at 150 ms from image onset, and in the trials with an image that was displayed for 310 ms, the TMS was administrated at 300 ms from image onset. The image was followed by a random-dot mask lasting 1 s. Then, the question “What did you see?” displayed on the monitor. Participants were required to provide a verbal response with an answer of positive, negative, or neutral image. The experimenter obtained the response by pressing a key on the computer. Following the reaction, the screen remained dark for 7 s, maintaining a time gap of over 10 s between pulses to prevent alterations in motor excitability caused by TMS itself.

### 2.6. Data Analysis

The behavioral component assessed in this experiment was accuracy on the emotion recognition task. The neurophysiological component measured the mean peak-to-peak MEP amplitude (mV) in every correct trial for each condition, represented as a ratio of the mean peak-to-peak MEP amplitude (mV) in the condition compared with the baseline (condition/baseline). Custom script in SIGNAL 6.0 was used to extract the peak-to-peak amplitudes of MEP. Trials meeting the criteria of having a correct verbal response and voluntary root mean square EMG within 2 SD of the mean in the 100 ms before the TMS pulse were included for further analysis. The normality of the data was assessed using the Shapiro–Wilk test. The results indicated that the TMS output intensity and MEP data were normally distributed, whereas accuracy was not. Hence, one-way ANOVA was performed for the TMS output intensity, with group (HTA, MTA, and LTA) as the between-subject factor, and three-way RM ANOVA was conducted for MEP ratios, with group as the between-subject factor and image type (positive, negative, and neutral) and TMS timing (150 vs. 300 ms) as within-subject factors. RM ANOVA was conducted with SPSS 22.0 (IBM Corp., Armonk, NY). For accuracy, aligned rank transformation analysis of variance (ART ANOVA), a nonparametric method that allows for repeated measures, was conducted with the ARTool in the RStudio [28, 29]. The Greenhouse–Geisser technique was employed to adjust for deviations from sphericity. If ANOVA revealed significant interaction effects, post hoc analysis was conducted using either paired samples *t*-tests, independent samples *t*-tests, or Wilcoxon signed rank test with Bonferroni correction. Finally, the Pearson correlation analysis was conducted for anxiety score and accuracy/MEP ratio. The significance was determined at *p* < 0.05. With an *α* level of 0.05, we estimated the statistical power of the outcome post hoc by using G*∗*Power 3.1 with the generic F-test model [30]. Data are shown as mean ± standard deviation.

## 3. Results

### 3.1. Demographic Information

The range of the trait anxiety in each group was also consistent with the inclusion criterion of previous studies [31, 32]. See Table 1 for the characteristics of each group. A one-way ANOVA was performed for trait anxiety score and demonstrated a significant main effect of the group (*F*_ (2,84)_ = 234.310, *p* < 0.001, *η*_p_^2^ = 0.848). Post hoc analysis showed a significant difference between each two groups (*ps*  < 0.001), which confirms the distribution of the scores for the participants selected into three distinct groups.

### 3.2. Behavioral Data

**Figure 2:**
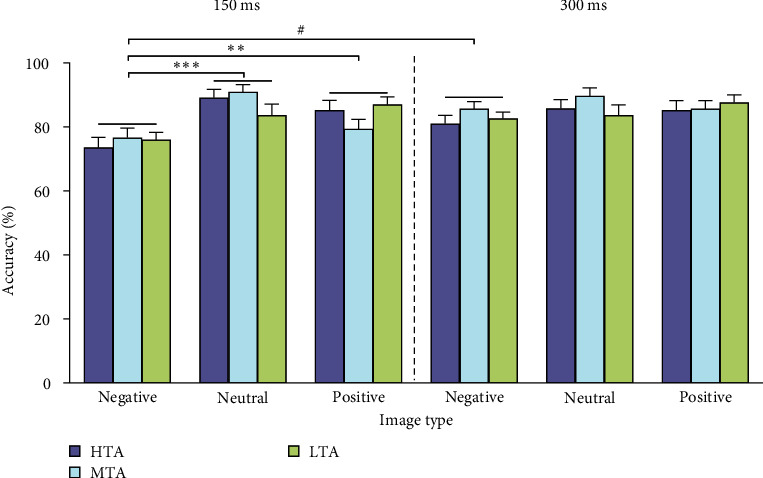
Accuracy across the three groups and image types. *⁣*^*∗∗*^*p* < 0.01 and *⁣*^*∗∗∗*^*p* < 0.001 for significant differences between the indicated image types among all groups. ^#^*p* < 0.05 for significant differences between time points within one image type. HTA, high trait anxiety; MTA, moderate trait anxiety; LTA, low trait anxiety.

The ART ANOVA demonstrated a significant main effect of image type (*F*_ (2, 420)_ = 23.763, *p* < 0.001, *η*_p_^2^ = 0.102) and TMS timing (*F*_ (1, 420)_ = 7.214, *p*=0.008, *η*_p_^2^ = 0.017). The interaction between image type and TMS timing was also significant (*F*_ (2, 420)_ = 4.595, *p*=0.011, *η*_p_^2^ = 0.021, Figure 2). Post hoc Wilcoxon signed rank test showed that when TMS was delivered at 150 ms, the accuracy for negative images was lower than that for both positive (*p*=0.003) and neutral images (*p* < 0.001). Post hoc test also showed that the accuracy for negative images when TMS was delivered at 150 ms was also lower than that at 300 ms (*p*=0.041). No other significant main effects or interactions were found.

### 3.3. Neurophysiological Data

**Figure 3:**
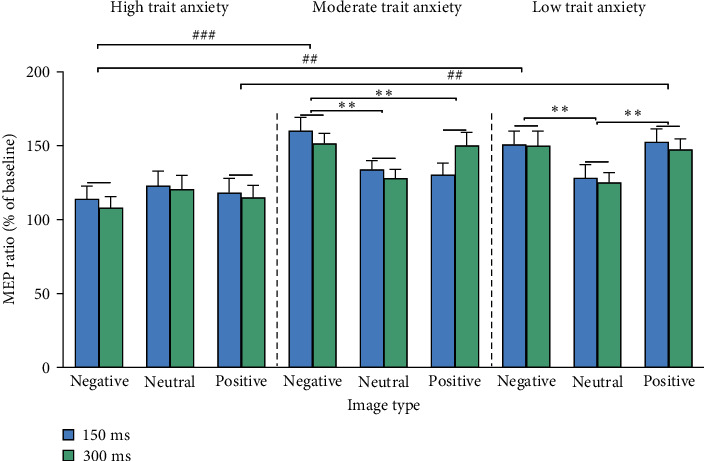
MEP ratios across the three groups and image types. *⁣*^*∗∗*^*p* < 0.01 for significant differences between the indicated image types within one participant group. ^##^*p* < 0.01 and ^###^*p* < 0.001 for significant differences between the indicated participant groups within one image type.

The mean TMS output intensity (intensity set at 120% of RMT) was 54.07% ± 8.85% of the maximum stimulation output for the HTA group, 50.70% ± 9.28% for the MTA group, and 49.40% ± 6.96% for the LTA group. One-way ANOVA performed for the mean TMS output intensity indicated that the main effect of the group was not significant (*F*_ (2,84)_ = 2.309, *p*=0.106, *η*_p_^2^ = 0.052). The baseline MEP amplitude was 1.00 ± 0.24 mV for the HTA group, 1.01 ± 0.17 mV for the MTA group, and 0.99 ± 0.22 mV for the LTA group. MEP ratios in emotion recognition task were shown in Figure 3. The RM ANOVA assessing MEP ratios demonstrated a significant main effect of the group (*F*_ (2, 84)_ = 5.571, *p*=0.005, *η*_p_^2^ = 0.117) and image type (*F*_ (1.688, 141.777)_ = 6.588, *p*=0.003, *η*_p_^2^ = 0.073). The interaction between group and image type was also significant (*F*_ (4, 168)_ = 6.124, *p* < 0.001, *η*_p_^2^ = 0.127); with a noncentrality parameter of 24.496, post hoc analyses indicated that the power (1−*β*) was 98.6%. Paired samples *t*-tests between different image types among each group showed that for the LTA group, the MEP ratios of both positive and negative images were significantly higher than that for neutral images (positive vs. neutral: *p*=0.001, 95% CI = [8.16, 38.57]; negative vs. neutral: *p*=0.004, 95% CI = [6.36, 41.25]). For the MTA group, the MEP ratio for negative images was significantly higher than those for both positive and neutral images (negative vs. positive: *p*=0.005, 95% CI = [3.90, 27.36]; negative vs. neutral: *p*=0.002, 95% CI = [7.49, 42.38]). No significant differences were found for MEP ratios among the image types in the HTA group (*ps*  > 0.05). In addition, independent samples *t*-tests between different groups among each image type showed that for positive images, the MEP ratios of HTA group were significantly lower than that of the LTA group (*p*=0.005, 95% CI = [−58.24, −8.49]); for negative images, the MEP ratios of the HTA group were significantly lower than that of both the MTA (*p* < 0.001, 95% CI = [−71.26, −18.47]) and LTA group (*p*=0.001, 95% CI = [−65.82, −13.04]). No other significant main effects or interactions were found.

### 3.4. Pearson Correlation

**Figure 4:**
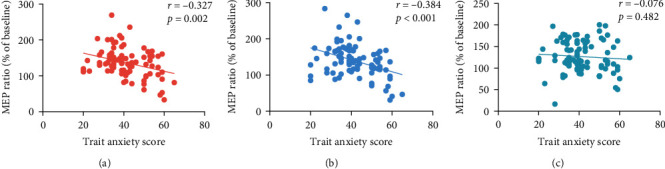
Correlation between the trait anxiety score and MEP ratios for each image types. (A) Positive image, (B) negative image, and (C) neutral image.

The Pearson correlation between trait anxiety score and accuracy showed no significant correlations (*ps*  > 0.05). The Pearson correlation between trait anxiety score and MEP ratios showed that trait anxiety score was significantly negative correlated with the MEP ratios for both positive images (*r* = −0.327, *p*=0.002) and negative images (*r* = −0.384, *p* < 0.001) but not neutral images (*r* = −0.076, *p*=0.482; Figure 4).

## 4. Discussion

The present study explored how increasing levels of anxiety affected the perception of different emotional stimuli by measuring cortical excitability during the perception of different emotional stimuli at two time windows. Our key findings were that trait anxiety modulated emotional perception at both the early and later time windows during the perception of emotional stimuli, with trait anxiety first diminishing the cortical excitability corresponding to emotional perception of positive stimuli and then to that of negative stimuli. Moreover, anxiety severity was associated with emotional perception–related cortical excitability.

Numerous studies have reported neuronal dysfunction in brain regions associated with emotion processing (e.g., the amygdala and hypothalamus) and cognitive processing (e.g., the frontal and parietal cortices) as well as an imbalance in the distributed functional network in individuals with HTA [5, 33, 34]. Motor systems have been widely reported to be not only involved in motor output but also engaged in emotion processing, with cortical excitability reflecting the degree of the motor system mobilization to launch adaptive motor responses corresponding to different emotional stimuli [10, 35]. Here, we showed that emotional perception–related cortical excitability may serve as a neurophysiological marker for identifying anxiety levels [36]. In the LTA group, cortical excitability during the perception of both positive and negative images was significantly higher at both 150 and 300 ms after stimulus onset, supporting the idea that motor networks within the right hemisphere were particularly involved during recognition of emotional stimuli [20]. Increased motor excitability at 150 ms is related to a rapid mobilization of the motor system for action preparation in response to observing the emotional stimulus, and increased motor excitability at 300 ms is directly linked to action regulation by the recognition of the specific emotional content conveyed by the stimulus [18, 20, 37].

For individuals in the MTA group, increased cortical excitability was detected only for negative stimuli not for positive stimuli. Both positive and negative stimuli could evoke automatic motor behavior (e.g., approaching positive stimuli and avoiding negative stimuli), and such social interaction is crucial in achieving many functional goals [38]. However, negative signals are particularly adept at inducing automatic evaluative reactions and rapid reactions since a deficit in the perception of negative emotion could lead to less successful detection and avoidance of potentially dangerous situations [39]. Hence, the present findings indicating that moderate anxiety affected only motor cortical excitability responses to positive emotion—and thus motor mobilization in response to negative stimuli was maintained for survival—supports the evolutionary thesis that increased motor preparation in the face of negative stimuli (e.g., a threat) may best ensure the survival of the organism [18].

As the level of anxiety increased, no specific emotion-driven motor excitability change was detected for either positive or negative stimuli in individuals with HTA, which may be explained by attenuated differential beta desynchronization responses to dynamic emotional stimuli [40]. Numerous studies have reported that individuals with HTA may have significant attentional bias to negative emotional stimuli [41, 42] as well as to positive stimuli [43]; however, difficulty in disengaging attention from these stimuli could be a barrier to launch action preparation, as reflected by diminished emotional perception–related cortical excitability.

The trait anxiety score was negatively correlated with the MEP ratio related to emotional perception, with higher trait anxiety scores associated with lower MEP ratios. Higher MEP ratios indicate a stronger mobilization of the motor system for action preparation, which may enable fast behaviors [44], such as approaching positive stimuli to induce greater joy or avoiding negative stimuli to protect one's self. However, those preponderant behavior tendencies were restrained in individuals with trait anxiety, suggesting that they are unable to perform adaptive behavior, which in turn, their anxiety was deepened [19].

Regarding accuracy, individuals with HTA have been found in some studies to be inferior at correctly recognizing emotional stimuli, especially negative stimuli [45, 46], whereas other studies have reported that there is no clear evidence of a relationship between trait anxiety and emotion recognition accuracy, as no differences in accuracy across seven emotional expressions were observed between persons with high vs. low levels of trait anxiety [47–49]. Here, we reported similar results, with no significant difference in accuracy for discriminating the emotion category among individuals with differing levels of anxiety severity. However, we found lower accuracy for recognition of negative images with TMS delivered at 150 ms than that at 300 ms, as well as lower than accuracy for recognition of positive and neutral images with TMS delivered at 150 ms (Figure 2). A previous study has shown that TMS applied over the right M1 at 150 ms from stimulus onset selectively interferes with the visual recognition of body expressions due to a lower accuracy in the early (150 ms) temporal condition relative to the late (300 ms) [19]. The authors explained that right M1 may reflect the spillover of somatosensory activity associated with emotion perception and that the decrease in accuracy could be due to the spread of TMS interference to closely interconnected right somatosensory regions, which in turn may affect emotion perception [19]. Here, we expanded this interference to the recognition of emotional contextual scenes and especially to negative information.

Our study has limitations. First, only 10 trials were collected per condition, which is similar to most M1 TMS studies. Even the recommendation of the International Federation of Clinical Neurophysiology is to use 8–10 trials per condition for M1 TMS studies [24]. However, more trials collected per condition would likely have provided more robust results. Second, baseline MEPs were collected only before the emotion recognition task, not after it. Recording baseline MEPs both before and after the task would have allowed for assessing whether the prolonged stimulation determined changes in motor cortical excitability over time. Third, during the emotion recognition task, future studies should consider monitoring whether the participants were paying attention during the task and whether they were looking at the image.

## 5. Conclusion

The findings of the present study indicated that individuals with different levels of trait anxiety have different emotional perception–related motor cortical excitability and that this effect was progressive with the anxiety level, with the motor cortical excitability responses to positive emotion decreased first and those to negative emotion decreased later. These findings have important psychological implications. Individuals with HTA experienced impaired motor cortical excitability responses to both positive and negative emotions, which may prevent them from launching appropriate actions in response to external emotional information, which in turn may further aggravate their anxiety.

## Figures and Tables

**Table 1:** Demographic information and mean scores for questionnaire measures of trait anxiety for three groups.

Group	*N* total (women)	Age (SD)	STAI trait anxiety (range)
High trait anxiety	27 (18)	20.1 (2.2)	54.3 (46–65)
Moderate trait anxiety	30 (22)	19.9 (2.1)	40.4 (36–44)
Low trait anxiety	30 (18)	20.7 (2.7)	30.6 (20–35)

## Data Availability

The data that support the findings of this study are available from the corresponding authors upon reasonable request.
